# Comparison between neoadjuvant and adjuvant gemcitabine plus cisplatin chemotherapy for muscle-invasive bladder cancer

**DOI:** 10.1111/ajco.12017

**Published:** 2012-11-06

**Authors:** Nobuaki Matsubara, Hirofumi Mukai, Yoichi Naito, Masahiko Nezu, Kuniaki Itoh

**Affiliations:** Division of Oncology and Hematology, National Cancer Center Hospital East, ChibaJapan

**Keywords:** adjuvant chemotherapy, bladder cancer, cisplatin, gemcitabine, neoadjuvant chemotherapy

## Abstract

***Aim***: Radical cystectomy plus platinum-based perioperative chemotherapy is a standard treatment for patients with clinically localized muscle-invasive bladder cancer. The standard perioperative chemotherapy is methotrexate, vinblastine, doxorubicin and cisplatin (MVAC). However, no prospective randomized trial has been published that compares neoadjuvant and adjuvant chemotherapy for bladder cancer. Moreover, the efficacy of perioperative chemotherapy with gemcitabine plus cisplatin (GC) has not been clarified. In this study we have compared the clinical outcomes between neoadjuvant and adjuvant chemotherapy in patients receiving GC.

***Methods***: We retrospectively reviewed the records of patients who were scheduled to be treated with a radical cystectomy plus perioperative chemotherapy with GC from 2005 to 2010 at our institution. The primary outcome measure was recurrence-free survival (RFS).

***Results***: A total of 42 patients received perioperative chemotherapy with GC (25 neoadjuvant, 17 adjuvant). The median number of cycles of GC administered to the two groups was not significantly different. The median duration of follow up was 28.6 months. During the follow-up period, recurrence was observed in nine and three patients in the neoadjuvant and adjuvant groups, respectively. The RFS rate at median follow up was 67 and 76% in the neoadjuvant and adjuvant groups, respectively. No significant difference in RFS at median follow up was observed between the two groups (*P* = 0.124).

***Conclusion***: Our results showed no statistically significant difference in RFS between neoadjuvant and adjuvant GC chemotherapy for muscle-invasive bladder cancer. We expect to validate these findings in a prospective randomized trial.

## Introduction

Radical cystectomy with pelvic lymph-node dissection is a standard treatment option in patients with clinically localized muscle-invasive bladder cancer.^1^,^2^ However, muscle-invasive bladder cancer has a high potential for systemic disease recurrence, which has been reported to occur in approximately 50% of patients during their clinical course, and then cause their death in almost all of them.^3^,^4^ Based on these observations, it is assumed that micrometastases already exist at the time of radical cystectomy. Traditionally, in order to avoid disease recurrence and death, many studies have tested the efficacy of perioperative systemic chemotherapy.^5^–^8^

The main rationale for early systemic chemotherapy (neoadjuvant) is to eradicate a micrometastasis and reduce the primary bladder tumor volume in order to facilitate the subsequent surgical procedure. For almost 20 years many platinum-based combinations of neoadjuvant chemotherapy have been explored.^2^,^5^,^9^–^13^ Several clinical trials have demonstrated improved survival benefits in patients who received neoadjuvant chemotherapy.^5^,^7^,^11^ A recent meta-analysis concluded that the addition of neoadjuvant platinum-based chemotherapy to a radical cystectomy provided a 5-year survival advantage of 5% on an additive scale.^14^,^15^ Based on the large body of this type of evidence, platinum-based neoadjuvant chemotherapy for patients with muscle-invasive bladder cancer is widely used in daily practice. However, neoadjuvant chemotherapy also includes definite potential disadvantages. It may, for example, delay the radical cystectomy and then cause some patients who cannot achieve a tumor response to become ineligible for receiving a radical cystectomy.

On the other hand, compared with neoadjuvant chemotherapy, adjuvant chemotherapy for bladder cancer has not provided as much strong evidence of a therapeutic response because of a lack of results from large randomized prospective trials. But adjuvant chemotherapy has already been accepted in daily practice to a certain degree. The main rationale for adjuvant chemotherapy administration is to reduce local and/or metastatic recurrence. The potential disadvantage of adjuvant chemotherapy is that it may cause a delay in micrometastasis eradication and that it may be impossible to administer a full dose of the chemotherapeutic agent due to surgical complications. A few small prospective and many retrospective studies have revealed a significant prolongation of relapse-free survival and the survival benefit in adjuvant chemotherapy.^16^–^18^ Based on the present weak evidence, adjuvant chemotherapy cannot be considered as a standard treatment option. Nevertheless, in daily practice, several patients, such as those with severe pollakiuria or hematuria, with renal functions that were inadequate for chemotherapy, have been treated with a radical cystectomy followed by adjuvant chemotherapy. In such cases, adjuvant chemotherapy is frequently utilized in daily practice. However, in order to become a standard treatment option, evidence that supports the equal efficacy of neoadjuvant and adjuvant chemotherapy might be needed. The equal efficacy of neoadjuvant and adjuvant chemotherapy has already been established in other malignancies such as breast cancer.^19^ However, in muscle-invasive bladder cancer very little evidence is available. No randomized trials have directly compared neoadjuvant with adjuvant chemotherapy. A few retrospective studies have been published but in those the chemotherapy regimen was not gemcitabine plus cisplatin (GC) but almost always methotrexate, vinblastine, doxorubicin and cisplatin.^20^

We hypothesized that the efficacies of neoadjuvant and adjuvant chemotherapy in patients undergoing a radical cystectomy might be equivalent. In order to test this hypothesis in this retrospective analysis, we reviewed and compared the outcomes, such as recurrence-free survival (RFS), in patients who received neoadjuvant or adjuvant GC chemotherapy at our institution.

## Methods

We retrospectively reviewed and analyzed the records of patients who were scheduled to undergo a radical cystectomy plus perioperative chemotherapy with GC at the National Cancer Center Hospital East (Kashiwa, Japan). The eligible patients were diagnosed as clinical stage T2–4, N0–2, M0 bladder cancer from April 2005 to December 2010. All patients were confirmed as having pathological muscle-invasive bladder cancer by transurethral resection. In this analysis, the pathological component was not limited to urothelial carcinoma; non-urothelial variants were allowed. Patients receiving other chemotherapy regimens and those with clinical stage < T2, with distant metastasis or with upper tract urothelial carcinoma, were excluded from this analysis. In principle, muscle-invasive or node positive disease was treated with neoadjuvant chemotherapy followed by a radical cystectomy in our institution. The patients who received preceding radical cystectomy followed by adjuvant chemotherapy had a reason for choosing this sequence, such as symptoms with a hematuria necessitating a cystectomy prior to chemotherapy or muscle-invasion of bladder discovered in the cystectomy specimen.

A radical cystectomy with pelvic lymph node dissection was performed by a urological surgeon at our institution. The pelvic lymph node dissection included the hypogastric, external iliac, obturator and distal common iliac lymph nodes. The patients who received a partial cystectomy (organ-sparing surgery) were also excluded from this analysis.

In principle, neoadjuvant or adjuvant chemotherapy was administered as four cycles of GC. Patients received gemcitabine 1000 mg/m^2^ on days 1, 8 and 15 plus cisplatin 70 mg/m^2^ on day 1 by i.v. infusion. The four cycles of chemotherapy were administered with 4-week intervals between the cycles. Prior to every chemotherapeutic agent infusion, the patients' laboratory data were assessed, such as kidney function by creatinine clearance and bone marrow function by white blood cell and thrombocyte counts. The dose of cisplatin in subsequent cycles was adjusted based on creatinine clearance. Gemcitabine administration was suspended in patients with white blood cell counts less than 2000/mm^3^, thrombocyte counts less than 5 × 10^4^/mm^3^ or other non-tolerable grade 3 or higher non-hematological toxicity on day 8 or 15.

In patients in the neoadjuvant setting, before starting chemotherapy, the bladder tumor was resected as completely as possible by TUR-BT. Interim analyses were performed after two cycles of chemotherapy by abdominal and pelvic computed tomography (CT) scan or magnetic resonance imaging (MRI). If no tumor response was identified, the neoadjuvant chemotherapy was stopped and then patients received a salvage cystectomy immediately. The clinical response after neoadjuvant chemotherapy was judged by the CT scan or MRI with the use of the response evaluation criteria in solid tumor (RECIST).

The primary outcome measure was RFS. The secondary outcome measures were a pathological complete response (pCR) rate and relative-dose intensity (RDI). All toxicity profiles were graded according to the National Cancer Institute common toxicity criteria for adverse events (CTC-AE) version 4.0. Treatment duration was determined from the start date of neoadjuvant chemotherapy administration to the date of the cystectomy in neoadjuvant patients and from the date of the cystectomy to the date of the final adjuvant chemotherapy administration in adjuvant patients. The length of follow up and start of survival analysis was determined from the start date of treatment to the date of recurrence confirmation or last follow up. Survival analysis was performed using the Kaplan–Meier method with patients stratified into neoadjuvant and adjuvant chemotherapy groups. A log–rank test was used to estimate and compare RFS among patients. Proportions were analyzed with the χ^2^ test. Continuous variables were analyzed with the Mann–Whitney *U*-test. All tests were two-sided. *P* < 0.05 was considered to be statistically significant. All analyses were performed according to the intention-to-treat principle. All data analyses were calculated using SPSW statistics 18.0 (SPSS, Chicago, IL, USA).

## Results

During the study period, 25 patients received neoadjuvant GC chemotherapy followed by a cystectomy and 17 patients received a cystectomy followed by adjuvant GC. In the adjuvant group, the reason for choosing adjuvant chemotherapy was as follow. Eight patients had symptoms with severe hematuria, seven patients had severe pollakiuria and muscle-invasion was discovered in the cystectomy specimen in two patients. Baseline clinical and pathological characteristics of these groups are summarized in Table 1. Comparing baseline characteristics of the two groups, significant differences were observed in sex (*P* = 0.016) and clinical node stage (*P* = 0.026). The neoadjuvant group was slightly older and at a lower clinical T stage than the adjuvant group, but this difference was not significant. No significant difference was observed among their other characteristics.

**Table 1:** Patients' characteristics

	Neoadjuvant	Adjuvant	*P*-value
Number of patients	25(%)	17(%)	
Age, years			
Mean	65	65	0.849
Median	67	64	0.877
Range	47–79	50–76	
Sex(*n*, %)			0.016
Male	15(60)	16(94)	
Female	10(40)	1(6)	
Clinical T stage(*n*, %)			0.391
≤cT2	9(36)	4(24)	
>cT2	16(64)	13(77)	
Clinical N stage(*n*, %)			0.026
cN0	16(64)	16(94)	
cN1 or 2	9(36)	1(6)	
Histology			0.298
Pure urothelial carcinoma	23(84)	13(77)	
Non-urothelial carcinoma or mixed component	4(16)	4(24)	

In principle, four cycles of GC chemotherapy were planned in both neoadjuvant and adjuvant settings. The mean and median numbers of cycles of GC between the two groups were not significantly different (neoadjuvant *vs* adjuvant; median 4.0 *vs* 4.0, *P* = 0.166; mean 3.80 *vs* 3.53, *P* = 0.073). Only one and two patients in the neoadjuvant and adjuvant groups, respectively, dropped out of chemotherapy due to non-tolerable toxicity. The hematological toxicity profiles of GC chemotherapy are listed in Table 2. The incidence rates of grade 3 or 4 thrombocytopenia, anemia and neutropenia were not significantly different between the two groups (data not shown). The RDI of gemcitabine and cisplatin showed a tendency to be higher in the neoadjuvant than in the adjuvant group but these differences were not statistically significant (neoadjuvant *vs* adjuvant; gemcitabine; 82 *vs* 78%; *P* = 0.460, cisplatin; 95 *vs* 82%; *P* = 0.073). The median treatment duration was 134 days for patients in the neoadjuvant group and 150 days for those in the adjuvant group. This difference in treatment duration between the two groups was statistically significant (*P* = 0.016). More details of the treatment procedure are shown in Table 3.

**Tabel 2:** Hematological toxicity of perioperative gemcitabine plus cisplatin regimen (*n*, %)

	Neoadjuvant (*n* = 25)			Adjuvant (*n* = 17)		
	Grade 1–2	Grade 3	Grade 4	Grade 1–2	Grade 3	Grade 4
	Number of patients (%)					
Anemia	17(68)	8(32)	0	15(88)	2(12)	0
Thrombocytopenia	14(56)	7(28)	3(12)	9(53)	3(17)	2(12)
Neutropenia	13(52)	7(28)	3(12)	8(47)	5(29)	1(5.8)
Febrile neutropenia	–	1(4)	1(4)	–	1(5.8)	0

**Table 3:** Treatment procedure

	Neoadjuvant	Adjuvant	*P*-value
Number of gemcitabine plus cisplatin cycles			
Median	4.0	4.0	0.166
Mean	3.80	3.53	0.073
Range	2–4	1–4	
Relative dose intensity (%)			
Gemcitabine	82	78	0.460
Cisplatin	95	82	0.073
Treatment duration, days			
Median	134.0	150.0	0.016
Mean	131.7	153.4	0.023
Range	62–195	94–246	
Follow-up, months			0.377
Median	25.1	33.8	
Range	3.4–58.4	6.8–64.9	

**Figure 1:**
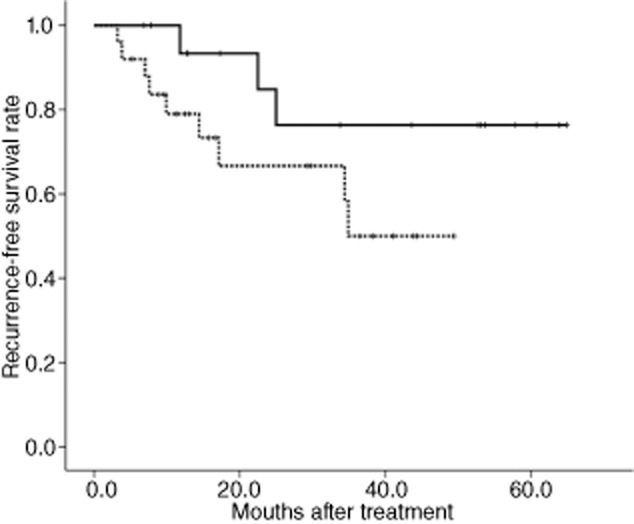
Disease-free survival curves stratified by patients on (----) neoadjuvant and (____) adjuvant chemotherapy. Log–rank *P* = 0.124.

The median follow-up period was 28.6 months in all patients (range; 3.4–64.9 months). The difference of median follow-up period between the two groups was not statistically significant (*P* = 0.377). During the follow-up periods, 12 patients (29%) experienced metastatic recurrences. Recurrence was observed in nine and three patients in the neoadjuvant and adjuvant groups, respectively. Kaplan–Meier curves of RFS stratified by treatment group are shown in Figure 1. The RFS rate at median follow up was 66.7 and 76%, in the neoadjuvant and adjuvant groups, respectively. No significant difference in RFS at median follow up was detected between the two groups (*P* = 0.124).

**Figure 2:**
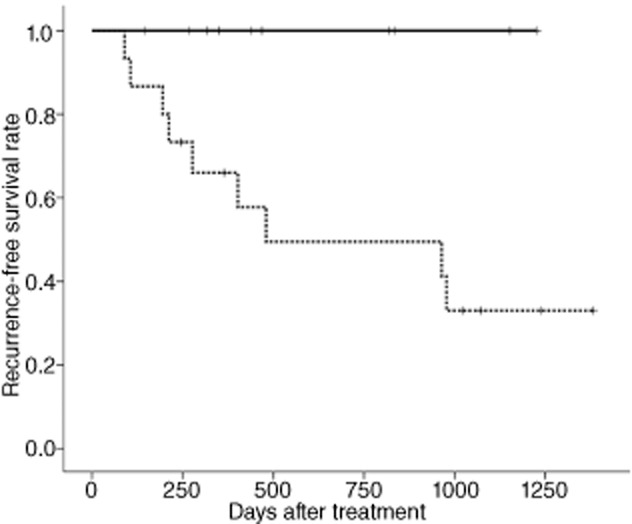
Recurrence-free survival curves stratified by (____) pathological complete response (pT0) and (----) non-pT0 in patients in neoadjuvant group. Log–rank *P* = 0.018.

The clinical and pathological responses in patients receiving neoadjuvant chemotherapy are shown in Table 4. The clinical response and complete response rates after neoadjuvant chemotherapy were 60% (15/25) and 44% (11/25), respectively. Three patients in the neoadjuvant group (12%) showed no tumor response after two cycles of neoadjuvant GC chemotherapy by radiological assessment and were then defined as having progressive disease (PD). The three patients who had experienced PD stopped neoadjuvant chemotherapy and underwent salvage cystectomy immediately. The pathological complete response (pT0) rate and downstaging (<pT2) rate after neoadjuvant chemotherapy were 40% (10/25) and 44% (11/25), respectively. None of the patients who achieved pT0 showed disease recurrence. Kaplan–Meier survival curves stratified by pT0 and non-pT0 in patients receiving neoadjuvant GC chemotherapy are shown in Figure 2. The RFS rates at median follow up were 100 and 50%, in patients with pT0 and non-pT0, respectively. The difference in RFS between these groups at median follow up was significant (*P* = 0.018). On the other hand, in the adjuvant group, no patient achieved pT0 in the cystectomy specimens.

**Table 4:** Clinical and pathological outcomes of neoadjuvant chemotherapy (*N* = 25)

	Number of patients (%)
Clinical outcome	
Complete response	11(44)
Partial response	4(16)
Stable disease	7(28)
Progressive disease	3(12)
Pathological outcome	
pT0	10(40)
pT1	1(4)
≥pT2	14(56)

pT, pathological complete response.

Positive pelvic node metastasis, which was analyzed in surgical specimens, was present in six patients in each of the neoadjuvant and adjuvant groups. The pathological node metastasis rates were not significantly different between the two groups (25 *vs* 35%; *P* = 0.49).

## Discussion

Perioperative systemic chemotherapy, especially neoadjuvant chemotherapy added to radical cystectomy, has been demonstrated in many randomized trials to provide a significant survival benefit.^5^,^7^ Based on this level I evidence, at our institution neoadjuvant chemotherapy followed by radical cystectomy has been increasingly used in patients with muscle-invasive bladder cancer. However, in almost all previous trials the chemotherapy regimen was not GC but other platinum-based combinations. To the best of our knowledge, studies with the GC regimen as neoadjuvant chemotherapy were performed only as minimal retrospective analyses.^21^,^22^ In addition, information about the usefulness of adjuvant chemotherapy is limited because of a lack of results from large randomized trials. No prospective studies or small retrospective analyses have directly compared neoadjuvant with adjuvant GC chemotherapy in patients with muscle-invasive bladder cancer. Despite the limited evidences of the benefit of adjuvant chemotherapy, especially the GC regimen, adjuvant chemotherapy already is widely used in daily practice.

This aim of this investigation was to compare the efficacy of the GC regimen in neoadjuvant and adjuvant settings. The results revealed no significant difference in RFS between the two modes of administration in patients with muscle-invasive bladder cancer. Our finding thus confirms two hypotheses. First, the efficacy of chemotherapy in neoadjuvant and adjuvant settings might be equivalent. Second, the GC regimen might be a candidate for perioperative standard chemotherapy. The result of the non-inferior RFS comparison between neoadjuvant and adjuvant administrations in this investigation is similar to that of the previous retrospective study.^20^ However, in the previous study the chemotherapy regimen was not limited to GC. All platinum-based chemotherapy regimens were allowed and the proportion of patients who received GC was only 35%. In addition, a subgroup analysis of the GC regimen in the previous study showed that the disease-specific survival was significantly worse in the adjuvant than in the neoadjuvant setting (hazard ratio 10.6; *P* = 0.049). This result was different from that of our study. Moreover, PFS curves were not significantly different but clearly separated in our study. There was some possibility that the *P*-value was not significant simply due to under-powering or a small sample size. There might be a number of confounding features and/or biases between the previous study and ours, such as patient selection, treatment decisions and RDI. Therefore, we cannot directly compare these results.

Other previous prospective trials have also supported the hypothesis of equal efficacy of the two modes of administration in perioperative chemotherapy. A trial at the University of Texas MD Anderson Cancer Center evaluated 140 patients who were randomized to a radical cystectomy with either five cycles of adjuvant MVAC or two cycles of neoadjuvant MVAC with three cycles of adjuvant MVAC. The result from this study demonstrated that the difference in disease-specific survival and overall survival was not significantly different between the two arms.^8^ However, the chemotherapy regimen in the MD Anderson trial also was not GC.

In our investigation there was no significant difference in the RDI of neoadjuvant and adjuvant GC administrations. According to this result, a potential disadvantage of adjuvant administration is eliminated by using neoadjuvant administration, such as surgical complications preventing administration of a full dose of the chemotherapeutic agent. Based on these results, including those of our investigation, the timing of perioperative chemotherapy is less important than whether or not patients actually receive it. Future prospective randomized trials (neoadjuvant *vs* adjuvant) of GC chemotherapy are thus warranted.

The efficacy equivalence of GC and MVAC has been generally accepted and GC has been adopted due to its being less toxic but the previous study was powered only to show superiority of GC and the results showed a trend towards equivalence.^23^ Better tolerability is an important factor in considering whether to introduce perioperative chemotherapy in daily practice. However, there have been no prospective trials reported of GC in neoadjuvant and/or adjuvant settings, but only a few retrospective studies.^21^,^22^ The tolerability gleaned from the RDI and the interruption rate seen in our investigation are satisfactory and similar to those seen in previous analyses. The concepts of RFS or disease-free survival are in general used as efficacy measurements in perioperative chemotherapy. The pCR may be relied on as a surrogate marker for efficacy measurement in a neoadjuvant setting for muscle-invasive bladder cancer. According to the subset analysis of the Southwest Oncology Group 8710 trial, the median overall survival in patients who achieved pT0 was 13.6 years.^5^ This duration is surprisingly long and may be regarded as a cure. From our results, no disease recurrence was observed in patients who achieved pT0. In addition, pCR appears to be a reasonable and suitable end-point for assessing the efficacy of various neoadjuvant chemotherapy regimens. The pCR rates were 38 and 32%, respectively, with MVAC in the SWOG8710 trial and with cisplatin, methotrexate and vinblastine in the Medical Research Council NC trial.^9^ In our investigation with GC, 40 and 44% of patients in the neoadjuvant setting achieved pCR and <pT2 at cystectomy, respectively. These results are almost the same as those of the previous trial with non-GC. Thus, the GC regimen seems to be suitable for neoadjuvant administration. However, various outcomes have been reported for the neoadjuvant GC, but the results are confusing. For example, Dash *et al*. used a 21-day schedule for four cycles of GC and reported that 26 and 36% of patients achieved pT0 and <pT2, respectively.^24^ The Cleveland Clinic used a standard administration of three cycles of GC and reported that only 7 and 31% of patients achieved pT0 and <pT2, respectively.^25^

There are several possible explanations for these varying and confusing results associated with the GC regimen. Major reasons may be that the planned neoadjuvant GC cycles were different in the studies and that the bladder tumor was resected by TUR-BT as completely as possible before neoadjuvant chemotherapy, which is the practice in some institutions including ours. These procedural differences might affect the pCR rate. In addition, there might be many minor reasons for the differences, such as baseline characteristics and RDI. However, a high pCR rate with four cycles of GC is impressive, and further studies are warranted to confirm this result.

The strength of our analysis may be summarized as follows: first, direct access to all medical records allowed for accurate documentation of all information. Second, none of our patients were lost to follow up. Third, a consistent chemotherapy drug delivery was assured due to patients being treated at single institution analysis, even though it was an off-protocol study. On the other hand, our study also has several limitations. The most important of these is that it is a retrospective study. Other limitations would be its small sample size and biases such as different baseline characteristics. However, we believe that our results might be useful in daily practice and for planning further prospective trials.

A review from the National Cancer Database with over 7000 patients demonstrated that adjuvant chemotherapy was used in 10% of patients, compared with 2% for neoadjuvant chemotherapy from 1998 to 2003.^26^ The reason why neoadjuvant chemotherapy is not generally used may be that the optimal chemotherapy cycles have not been definitely established. Further trials are needed to determine the optimal cycles for both neoadjuvant and adjuvant chemotherapy. In fact, further prospective randomized trial of neoadjuvant and adjuvant chemotherapy are expected, but they seem to be impossible for number of reasons, such as need for a huge sample size and the unethical deviation from level 1 evidence of treatment not with neoadjuvant chemotherapy but with adjuvant chemotherapy for muscle-invasive bladder cancer. I believe a large sample size and many retrospective studies are the best we can get. In addition, new methodological approaches, such as determining the p53 status and gene expression profile to establish chemo-sensitivity, may be expected to be introduced in daily practice in the near future.

In conclusion, our results demonstrated there was no significant difference in RFS between neoadjuvant and adjuvant GC chemotherapy. We expect to validate these findings in a prospective randomized trial.
